# Frailty (mFI-5) and Age Predict Medical Complications After Posterior Lumbar Interbody Fusion in Older Adults: A Retrospective Cohort Study

**DOI:** 10.3390/jcm15082847

**Published:** 2026-04-09

**Authors:** Jong-Hoon Jung, Jong-Hwan Hong, Ji-Ho Jung, Moon-Soo Han, Jung-Kil Lee

**Affiliations:** Department of Neurosurgery, Chonnam National University Hospital and Medical School, Gwangju 61469, Republic of Korea; ir21076@cnuh.com (J.-H.J.); ir16093@cnuh.com (J.-H.H.); ns00248@cnuh.com (J.-H.J.);

**Keywords:** frailty, aged, spinal fusion, lumbar vertebrae, postoperative complications, risk assessment

## Abstract

**Objective**: To evaluate the prognostic utility of the five-item modified Frailty Index (mFI-5) for postoperative outcomes in older adults undergoing posterior lumbar interbody fusion (PLIF) for degenerative lumbar disease and to develop an interpretable preoperative risk model for medical complications (CxME). **Methods**: We retrospectively reviewed consecutive patients aged ≥65 years who underwent PLIF for lumbar spondylosis at a single tertiary institution. Baseline demographics, comorbidities, symptoms, American Society of Anesthesiologists (ASA) physical status, bone mineral density, antithrombotic use, perioperative laboratory findings, and operative variables were collected. CxME were defined as Clavien–Dindo grade ≥ II complications occurring during index hospitalization or within 30 days postoperatively. mFI-5 was calculated from five preoperative variables and stratified as 0, 1, and ≥2. Multivariable logistic regression was used to identify independent predictors of CxME. **Results**: Among 255 patients (mean age 72.6 years), 53 (20.8%) developed CxME. Patients with CxME were older and had higher rates of diabetes mellitus and preoperative dependency. mFI-5 ≥ 2 was more frequent in patients with CxME than in those without (66.0% vs. 27.3%, *p* < 0.001). Higher frailty was associated with older age, greater comorbidity burden, higher ASA class, lower preoperative hemoglobin, greater transfusion exposure, longer hospital stay, and a higher incidence of CxME (14.0%, 9.6%, and 38.9% for mFI-5 scores 0, 1, and ≥2, respectively; *p* < 0.001). In multivariable analysis, age (OR 1.133, 95% CI 1.055–1.216; *p* < 0.001) and mFI-5 (OR 2.103, 95% CI 1.387–3.188; *p* < 0.001) independently predicted CxME. The Age + mFI-5 model showed fair discrimination (optimism-corrected area under ROC 0.734). **Conclusions**: Preoperative frailty and age independently predicted postoperative medical complications after PLIF in older adults. The Age + mFI-5 model may support risk stratification, counselling, and perioperative optimization.

## 1. Introduction

The Republic of Korea, like many industrialized nations, is experiencing a significant demographic shift characterized by a steadily increasing proportion of its elderly population. This demographic trend directly contributes to an age-adjusted rise in the incidence of degenerative spinal diseases, resulting in a rapidly growing number of elderly patients requiring surgical intervention for degenerative lumbar conditions [1]. Consequently, as the proportion of elderly surgical candidates continues to expand, it becomes imperative to transcend a singular focus on chronological age when assessing surgical risks. Instead, a more comprehensive evaluation necessitates the integration of factors such as frailty and other patient-specific characteristics to enhance the accuracy of surgical risk stratification and optimize postoperative outcomes.

Frailty, a multifaceted geriatric syndrome characterized by age-associated declines in physiological reserve and function across multiple organ systems, significantly escalates vulnerability to adverse health outcomes. Among the various instruments employed to quantify frailty, the 11-item modified frailty index (mFI-11) has emerged as a widely recognized and robust predictor of postoperative complications and unfavourable outcomes following surgical treatment for degenerative spinal disease [2]. While the utility of mFI-11 in evaluating prognosis across diverse surgical disciplines is well-established, an increasing body of research highlights the comparable efficacy of the 5-factor modified frailty index (mFI-5). The mFI-5, offering a simplified calculation derived from a more concise set of five variables, is increasingly demonstrated to be an effective and practical tool for predicting prognosis in surgical populations [3,4].

Posterior lumbar interbody fusion (PLIF) is a well-established surgical technique for the management of degenerative spinal disease. Its efficacy stems from a direct posterior approach that facilitates thorough decompression of neural elements by enabling the effective removal of compressive posterior structures, thereby affording excellent intraoperative visualization [5,6]. In the geriatric population presenting with degenerative lumbar pathologies, often accompanied by segmental instability or refractory discogenic pain, PLIF is frequently indicated when conservative measures have failed. Given the increased physiological vulnerability and prevalence of comorbidities in elderly patients, the ability to predict the perioperative course and postoperative outcomes has become a critical component of contemporary spinal care. Consequently, there is a growing emphasis on comprehensive preoperative risk stratification to optimize patient selection and mitigate potential adverse events.

While multiple studies have established a significant association between frailty and adverse surgical outcomes across various spinal fusion techniques, employing different frailty indices as prognostic tools [7,8], a notable gap persists in the literature. Specifically, there is a paucity of research investigating the predictive utility of the mFI-5 for outcomes following PLIF surgery in the geriatric population. To address this deficiency, the present study was designed to evaluate the sufficiency of the mFI-5 as a risk assessment tool exclusively in patients aged 65 and older undergoing PLIF for degenerative lumbar disease. This investigation further aims to identify specific factors that influence the incidence of postoperative complications and overall surgical outcomes within this patient cohort.

## 2. Materials and Methods

### 2.1. Patient Selection and Data Collection

This study was designed and reported as a retrospective single-centre cohort study in accordance with the STROBE statement. We retrospectively identified consecutive patients aged ≥65 years who underwent posterior lumbar interbody fusion (PLIF) for lumbar spondylosis at a single tertiary institution between January 2013 and February 2023 and who had at least 30 days of postoperative follow-up. All consecutive eligible patients during the study period were included. Cases involving long-segment instrumentation for deformity correction and those performed for trauma, tumour, or spinal infection were excluded.

All PLIF procedures were performed by three experienced spine surgeons using a standardized technique consisting of bilateral laminectomy and facetectomy, bilateral discectomy, insertion of two interbody cages, and bilateral transpedicular screw fixation. Clinical data were extracted from the electronic medical record. Demographic and baseline variables included age, sex, body mass index (BMI), bone mineral density (BMD), comorbidities, main symptoms and symptom duration, medical history, surgical history, and American Society of Anesthesiologists (ASA) physical status.

Operative variables included surgical extent, operative time, estimated blood loss, transfusion status, incidental dural tear, and perioperative hemoglobin change based on preoperative and postoperative values. Postoperative surgical outcomes included new neurological deficit, cerebrospinal fluid leakage, surgical site infection, hardware failure, and reoperation. Length of stay (LOS) and unplanned readmission within 30 days after discharge were also recorded.

Postoperative medical complications were classified according to the Clavien–Dindo system, in which grade I represents minor deviations from the normal postoperative course not requiring pharmacological treatment or procedural intervention; grade II requires pharmacological treatment; grade III requires surgical, endoscopic, or radiological intervention; grade IV denotes life-threatening complications requiring intensive care; and grade V indicates death. In this study, medical complications were defined as Clavien–Dindo grade II or higher events occurring during the index hospitalization or within 30 days postoperatively. These events were recorded both as presence/absence and by specific complication type. Grade I events were excluded because they are often minor and self-limited, whereas grade II or higher events more clearly reflect complications requiring active medical management. The list of complications is provided in Supplementary Table S2.

We selected this composite endpoint to capture the full burden of early perioperative medical morbidity, recognizing that clinically relevant complications in older adults may occur either before discharge or shortly thereafter during the immediate post-discharge recovery period. Follow-up data from outpatient clinic visits were comprehensively reviewed. Owing to the retrospective single-centre design, complications managed outside our institution may have been underascertained. The study protocol was approved by the institutional review board (IRB No. CNUH-2023-398).

### 2.2. mFI-5 Score

The 5-item modified frailty index (mFI-5), a simplified derivative of the original mFI-11 developed from the American College of Surgeons National Surgical Quality Improvement Program (ACS NSQIP), was used for risk stratification (Table 1). The mFI-5 score for each patient was calculated based on documented comorbidities and preoperative functional status. The five components were: (1) hypertension requiring medication, (2) insulin-dependent or non-insulin-dependent diabetes mellitus, (3) history of chronic obstructive pulmonary disease or pneumonia, (4) history of congestive heart failure within 30 days before surgery, and (5) partially or totally dependent preoperative functional health status at the time of surgery.

Each component was assigned one point if present, yielding a total score ranging from 0 to 5. Patients were subsequently stratified into three groups for analysis: no frailty (mFI-5 = 0), mild frailty (mFI-5 = 1), and moderate-to-severe frailty (mFI-5 ≥ 2).

### 2.3. Statistical Analysis

All statistical analyses were performed using Python (version 3.9) with standard scientific libraries, including pandas, NumPy, SciPy, statsmodels, and scikit-learn. Continuous variables are presented as mean ± standard deviation and were compared using Student’s *t*-test or one-way analysis of variance (ANOVA), as appropriate. For non-normally distributed variables, the Mann–Whitney U test or Kruskal–Wallis test was used. Categorical variables are presented as frequencies and percentages and were compared using the chi-square test or Fisher’s exact test, depending on expected cell counts. There were no missing data for the variables included in the analysis.

The association between frailty and age was assessed using Pearson’s correlation coefficient for linear relationships and Spearman’s rank correlation coefficient for monotonic relationships. Multicollinearity among covariates was evaluated using the variance inflation factor (VIF), with VIF values < 5 considered indicative of negligible multicollinearity. Statistical significance was defined as a two-sided *p*-value < 0.05.

### 2.4. Model Development and Internal Validation

The primary objective of the modelling analysis was to develop a simple preoperative risk-stratification model for postoperative medical complications. Accordingly, the primary model was prespecified to include only age and mFI-5, as both variables are available before surgery and are readily applicable in routine clinical decision making. Because 53 CxME events occurred, the events-per-variable ratio was 26.5 in the primary two-predictor model. We intentionally adopted a parsimonious modelling strategy to enhance clinical interpretability and reduce the risk of overfitting.

No automated stepwise variable-selection procedure was used, and predictors were not screened solely on the basis of univariable *p*-values. Instead, predictors were selected a priori according to clinical relevance and intended model use. Transfusion was analyzed separately as an exploratory perioperative covariate rather than as part of the final preoperative prediction model, because it is not known at the time of preoperative risk assessment.

Multivariable logistic regression was performed to identify independent predictors of medical complications, and results are reported as odds ratios (ORs) with 95% confidence intervals (CIs). Individual-level predicted probabilities were calculated from the fitted model and used for subsequent discrimination, calibration, and visualization analyses.

To address potential overfitting, internal validation was performed using bootstrap resampling with 1000 iterations. For each bootstrap sample, the model was refitted and then evaluated in both the bootstrap sample and the original dataset. The average optimism was estimated for the area under the receiver operating characteristic curve (AUROC), calibration intercept, and calibration slope, and these values were used to derive optimism-corrected performance estimates.

Model discrimination was evaluated using receiver operating characteristic (ROC) analysis. Sensitivity and 1 − specificity were calculated across all possible probability thresholds, and the ROC curve was constructed by plotting sensitivity against 1 − specificity. To derive an interpretable classification threshold from the continuous predicted probabilities, we used the Youden index, defined as sensitivity + specificity − 1. The probability cut-off that maximized the Youden index was selected as the optimal threshold, as it represents the point on the ROC curve that provides the best overall balance between sensitivity and specificity.

Model calibration was assessed using both graphical and quantitative methods. Predicted probabilities were partitioned into deciles, and for each decile, the mean predicted probability and observed complication rate were calculated. A calibration plot was generated by plotting observed event rates against mean predicted probabilities, with the 45° line representing perfect calibration. The resulting probability matrix was also displayed as a heatmap. In addition, calibration-in-the-large (intercept), calibration slope, and the Brier score were calculated to quantify agreement between predicted and observed risk.

## 3. Results

### 3.1. Demographics

Of the 255 patients included, 53 (20.8%) developed at least one medical complication (CxME), whereas 202 (79.2%) did not. No patients were lost to follow-up for the primary endpoint. In demographic comparisons, patients with medical complications were significantly older than those without complications (75.23 ± 4.59 vs. 71.92 ± 4.70 years; *p <* 0.001). Sex distribution (male:female, 27:26 vs. 87:115; *p* = 0.305), BMI (24.73 ± 3.76 vs. 24.62 ± 3.08 kg/m^2^; *p* = 0.985), current smoking (7.5% vs. 6.4%; *p* = 0.760), and alcohol use (5.7% vs. 9.9%; *p* = 0.428) were comparable between groups (Table 2).

Regarding baseline clinical characteristics, diabetes mellitus was more frequent in the complication group (49.1% vs. 22.8%; *p <* 0.001), and preoperative dependency was also higher among patients who developed medical complications (28.3% vs. 13.9%; *p* = 0.012). In contrast, hypertension (79.2% vs. 70.3%; *p* = 0.196), dyslipidemia (18.9% vs. 12.9%; *p* = 0.264), chronic kidney disease (3.8% vs. 3.5%; *p* = 1.000), COPD (5.7% vs. 2.5%; *p* = 0.369), coronary artery disease (13.2% vs. 14.4%; *p* = 0.831), and congestive heart failure (3.8% vs. 1.0%; *p* = 0.192) did not differ significantly. Preoperative hemoglobin levels, as well as the use of antiplatelet or anticoagulant therapy, were not significantly associated with the occurrence of CxME.

Frailty burden differed markedly between groups. The distribution of mFI-5 scores was shifted towards higher values in patients who developed CxME (*p <* 0.001). Specifically, in the no-complication group, mFI-5 scores were 0 in 43 patients (21.3%), 1 in 104 (51.5%), 2 in 46 (22.8%), and 3 in 9 (4.5%). In contrast, in the complication group, mFI-5 scores were 0 in 7 patients (13.2%), 1 in 11 (20.8%), 2 in 27 (50.9%), and 3 in 8 (15.1%). When dichotomised, an mFI-5 score ≥ 2 was present in 66.0% (35/53) of patients with CxME versus 27.3% (55/202) without CxME, indicating a substantially higher frailty burden among those who experienced medical complications. In contrast, the ASA physical status distribution did not differ significantly between the groups.

There was no between-group difference in the number of fused levels. In contrast, perioperative transfusion was significantly more frequent in the CxME group (15/53, 28.3%) than in the non-CxME group (24/202, 11.9%; *p* = 0.005). Moreover, surgical site infection occurred more often in patients with CxME (7/53, 13.2%) than in those without CxME (5/202, 2.5%; *p* = 0.004), and reoperation was also higher in the CxME group (7/53, 13.2% vs. 9/202, 4.5%; *p* = 0.028). There were three deaths, all of which were attributable to medical complications; deaths occurred within the 30-day postoperative window and were classified as CxME (Clavien–Dindo grade V).

### 3.2. Subgroup Analysis According to mFI-5

Patients were stratified into three frailty groups: mFI-5 = 0 (*n* = 50), mFI-5 = 1 (*n* = 115), and mFI-5 ≥ 2 (*n* = 90) (Table 3). Increasing frailty was associated with a stepwise increase in age (71.14 ± 4.74, 72.56 ± 4.72, and 73.49 ± 4.94 years, respectively; *p* = 0.029), while sex distribution and BMI did not differ significantly across groups. Preoperative comorbidity burden rose markedly with higher mFI-5. The prevalence of hypertension (0.0%, 85.2%, and 95.6%), diabetes mellitus (0.0%, 5.9%, and 72.2%), dyslipidemia (6.0%, 11.3%, and 22.2%), chronic kidney disease (0.0%, 1.7%, and 7.8%), COPD (0.0%, 0.0%, and 8.9%), coronary artery disease (2.0%, 13.0%, and 22.2%), and congestive heart failure (0.0%, 0.0%, and 4.4%) differed significantly across frailty strata (all *p* ≤ 0.024). In contrast, cerebrovascular accident and tuberculosis histories did not vary by frailty group.

Patients with higher mFI-5 also had worse baseline status and differing perioperative exposures: ASA physical status increased across groups (*p <* 0.001), while preoperative hemoglobin was lower with increasing frailty (13.41 ± 1.29, 12.98 ± 1.27, 12.69 ± 1.59 g/dL; *p* = 0.016). Antiplatelet agent exposure and anticoagulant use increased with frailty (antiplatelet: 5.9%, 20.9%, 36.0%; anticoagulant: 2.0%, 1.7%, 13.3%; both *p <* 0.001), and dependency was substantially more common in the highest frailty group (0.0%, 13.0%, 23.6%; *p <* 0.001). Femoral neck and spine BMD did not differ significantly across frailty categories.

Surgery-related variables were largely similar across frailty groups in terms of surgical extent and operative time: the distribution of fusion levels did not differ, and operative time was comparable (258.20 ± 56.20, 266.43 ± 65.35, 258.67 ± 59.54 min; *p* = 0.788). However, transfusion exposure increased with frailty (5.9%, 13.0%, 23.6%; *p* = 0.007). Estimated blood loss differed across groups (374.24 ± 205.44, 384.44 ± 299.04, 317.01 ± 284.77 mL; *p* = 0.046), whereas hemoglobin drop showed a non-significant trend.

Postoperatively, the incidence of medical complications increased substantially with higher frailty (14.0%, 9.6%, and 38.9%; *p <* 0.001), paralleled by higher Clavien–Dindo classification scores (0.28 ± 0.70, 0.22 ± 0.72, 0.89 ± 1.23; *p <* 0.001). Length of stay also rose across frailty groups (11.66 ± 8.31, 14.36 ± 15.89, 16.70 ± 20.56 days; *p <* 0.001). In contrast, surgical site infection, surgical complications, reoperation, readmission, and mortality did not differ significantly by mFI-5 category.

### 3.3. Multivariable Logistic Regression for Medical Complications

**Figure 1:**
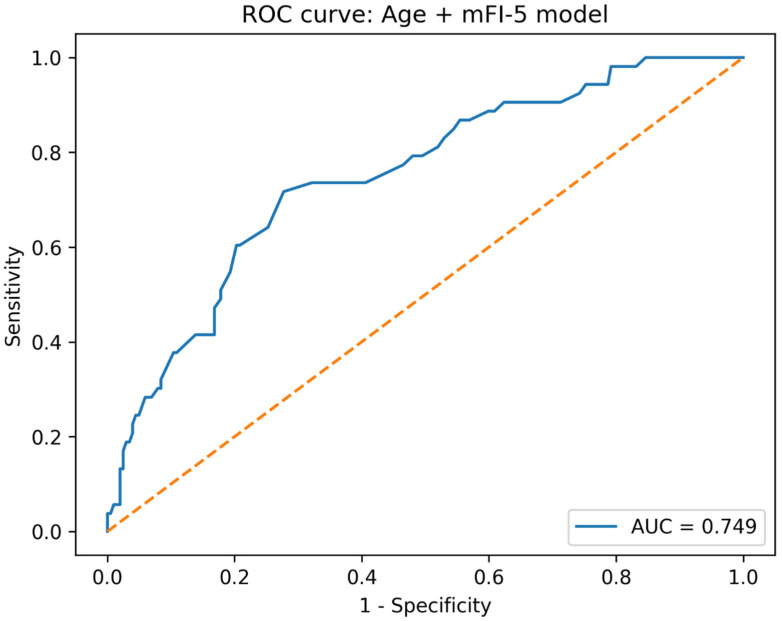
Receiver operating characteristic (ROC) curve demonstrating the discriminative performance of the preoperative Age + modified Frailty Index-5 (mFI-5) logistic regression model for predicting postoperative medical complications (CxME). The area under the curve (AUC) is 0.749, indicating favourable discrimination.

**Figure 2:**
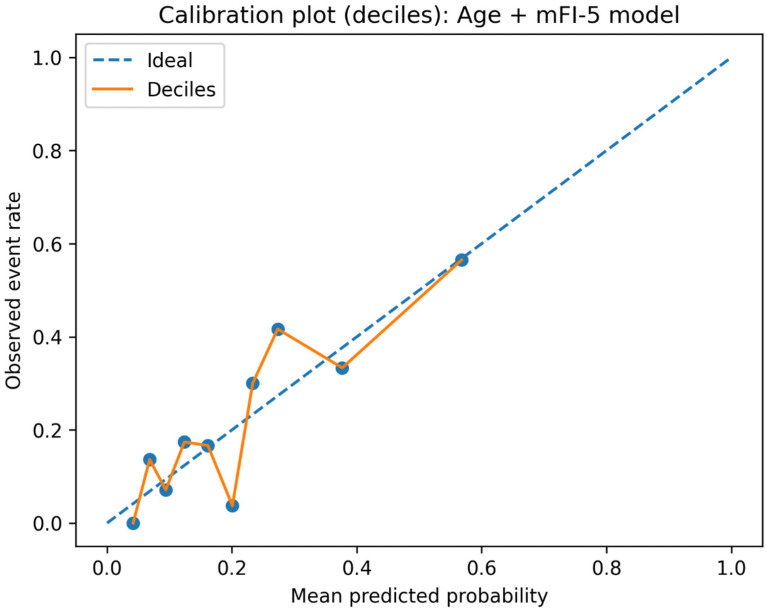
Calibration plot comparing predicted and observed probabilities of postoperative medical complications (CxME) for the Age + modified Frailty Index-5 (mFI-5) logistic regression model. Predicted risks are grouped into deciles, with observed event rates plotted against mean predicted probabilities. The diagonal line represents perfect calibration. Observed event rates increase with higher predicted probabilities across risk strata, indicating acceptable calibration.

**Figure 3:**
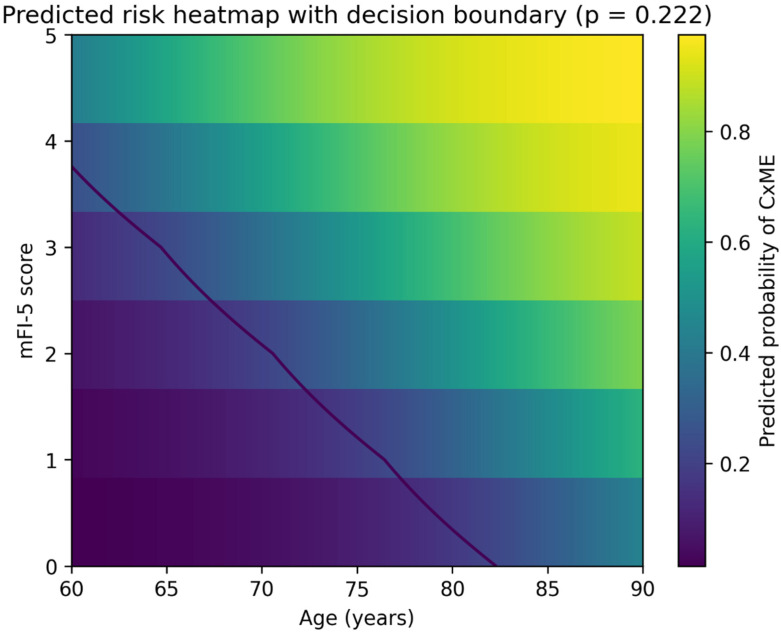
Heatmap showing the predicted probability of postoperative medical complications (CxME) across combinations of age and modified Frailty Index-5 (mFI-5) based on the Age + mFI-5 logistic regression model. Warmer colours indicate higher predicted risk. The contour line represents the Youden-optimal probability threshold (*p* = 0.222).

In multivariable logistic regression analysis (Table 4), older age and a higher mFI-5 score were independently associated with the occurrence of CxME. Specifically, each 1-year increase in age was associated with higher odds of CxME (OR 1.133, 95% CI 1.055–1.216, *p <* 0.001), and each 1-point increase in mFI-5 was also associated with higher odds (OR 2.103, 95% CI 1.387–3.188, *p <* 0.001). Logit(p) = −12.0979 + 0.1323 × Age + 0.7464 × mFI-5. In a separate exploratory perioperative-adjusted model, transfusion showed a positive but non-significant association after adjustment for age and mFI-5, suggesting that transfusion may reflect perioperative course severity rather than serve as a core preoperative predictor. The discriminative performance of the preoperative Age + mFI-5 model is shown in Figure 1. The model demonstrated favourable discrimination, with the area under ROC of 0.749 (bootstrap 95% CI 0.675–0.820). After bootstrap internal validation with 1000 resamples and optimism correction, the area under ROC was 0.734, indicating only modest optimism. Calibration was also preserved, with an optimism-corrected calibration intercept of 0.007 and calibration slope of 0.962, suggesting minimal overall miscalibration and limited overfitting. The apparent Brier score of the final Age + mFI-5 model was 0.142, indicating acceptable overall predictive accuracy and improved performance compared with the null model based on the event rate alone. Using the Youden-optimal probability threshold (*p* = 0.222; Supplementary Table S1), sensitivity was 0.717, and specificity was 0.723, respectively. Calibration of the age + mFI-5 model is presented in Figure 2. When predicted risks were grouped into deciles, the observed event rates increased with higher predicted probabilities, indicating that the model provided risk stratification with broadly concordant predicted and observed trends across the risk spectrum. Figure 3 visualizes the predicted probability of CxME across combinations of age and mFI-5. The heatmap illustrates a monotonic increase in predicted risk with both increasing age and increasing mFI-5. The contour line denotes the Youden-optimal decision boundary (*p* = 0.222), demonstrating the trade-off between chronological age and frailty burden: for example, the predicted risk reached *p* = 0.222 at approximately ≥83 years when mFI-5 = 0, whereas the boundary shifted to younger ages with increasing mFI-5 (approximately ≥76 years for mFI-5 = 1, ≥71 years for mFI-5 = 2, and ≥65 years for mFI-5 = 3), highlighting the complementary contributions of age and frailty burden to medical-complication risk.

## 4. Discussion

The concept of frailty has become increasingly important in perioperative risk assessment for the growing geriatric surgical population. Distinct from chronological age, frailty reflects reduced physiological reserve and heightened vulnerability to perioperative stressors, and it has consistently been associated with adverse postoperative outcomes across surgical specialties [9,10,11]. In spine surgery—where degenerative pathology predominates in older adults—frailty may be particularly informative because postoperative recovery depends not only on surgical success but also on functional reserve, mobility, and the ability to withstand systemic complications.

The original mFI-11 has been widely used in surgical outcome research and provides a broader comorbidity-based frailty profile. However, the mFI-5 was developed as a simplified derivative with fewer variables and greater feasibility for routine clinical use and retrospective chart abstraction. From a practical standpoint, this simplification may enhance bedside applicability without substantially compromising clinical utility. Nevertheless, our study was not designed as a head-to-head comparison between the mFI-11 and mFI-5. Therefore, while our findings support the mFI-5 as a pragmatic and clinically useful frailty tool in older PLIF patients, they should not be interpreted as definitive evidence that the mFI-5 fully replaces the mFI-11 in all settings.

A conceptual distinction should also be acknowledged when interpreting the present findings. Although the mFI-5 is widely used as a frailty measure in surgical outcome research, it is not a comprehensive multidimensional frailty phenotype. Rather, it is better regarded as a pragmatic, comorbidity-based surrogate of baseline vulnerability that incorporates a limited number of preoperative deficits, including functional dependence. Consequently, it captures an important component of perioperative risk, but does not directly measure other geriatric domains such as sarcopenia, mobility performance, cognitive reserve, nutritional status, or social vulnerability. Our findings therefore should not be interpreted as demonstrating that frailty in its fullest biological sense was comprehensively assessed. Instead, they indicate that the vulnerability represented by the mFI-5 is meaningfully associated with early postoperative medical complications in older adults undergoing PLIF.

In this study of patients aged ≥65 years undergoing PLIF for degenerative lumbar disease, frailty quantified by the mFI-5 demonstrated a strong relationship with postoperative medical complications. In multivariable analysis, both age and mFI-5 were independently associated with CxME, whereas transfusion showed a positive but non-significant association after adjustment. These findings support the interpretation that baseline vulnerability, captured by age and frailty burden, is a primary determinant of medical complications, whereas transfusion may function more as a perioperative course marker reflecting surgical or physiologic stress rather than as a consistent independent predictor once baseline risk is taken into account.

The subgroup analysis by mFI-5 provided additional clinical context for these associations. Increasing frailty was accompanied by a stepwise rise in comorbidity burden and worse baseline status, including higher ASA physical status, lower preoperative hemoglobin, greater use of antithrombotic agents, and a markedly higher prevalence of preoperative dependency. Importantly, surgery-related characteristics such as operative time and extent of fusion did not substantially differ by frailty category, suggesting that the observed differences in outcomes were not primarily driven by more extensive procedures in frailer patients. Nevertheless, transfusion exposure increased with higher frailty strata, consistent with reduced physiological reserve and/or a lower tolerance for perioperative anemia among medically complex patients.

Clinically, the most consequential outcome signal was the sharp increase in medical complications in the moderate-to-severe frailty group (mFI-5 ≥ 2), which approached 40%. This pattern reinforces the utility of mFI-5 for identifying older PLIF candidates at elevated risk for non-surgical sequelae. In contrast, the rates of surgical site infection, reoperation, and readmission did not demonstrate clear gradients across frailty strata in our subgroup analysis, likely reflecting low event counts for some endpoints and the fact that mFI-5 is primarily a measure of systemic vulnerability rather than local surgical risk. Notably, while SSI and reoperation were more frequent among patients who developed CxME in the overall cohort, this co-occurrence should be interpreted cautiously, as it may represent clustering of adverse postoperative events within the same patients rather than a straightforward causal pathway.

Frailty also showed implications for healthcare utilization. Length of stay increased significantly with higher mFI-5 categories, consistent with the expectation that frailer patients experience a more complex postoperative course and require longer recovery periods or additional medical management [12,13]. However, this finding should be interpreted cautiously. The absolute between-group difference may reflect not only complication burden and slower recovery in frailer patients but also discharge planning, rehabilitation needs, and other non-surgical institutional factors. Therefore, LOS should be viewed as a supportive secondary outcome rather than a primary driver of the study’s conclusions. From a perioperative planning standpoint, these findings support incorporating mFI-5 into preoperative counselling, discharge planning, and resource allocation, including early geriatric or medical co-management, rehabilitation planning, and enhanced monitoring pathways. From a practical perspective, routine preoperative identification of patients with mFI-5 ≥ 2 may help clinicians prioritize intensified medical optimization, closer postoperative monitoring, and more proactive discharge planning in elderly patients considered for PLIF.

We chose a composite endpoint encompassing complications during the index hospitalization and within 30 days postoperatively in order to capture the full burden of early perioperative medical morbidity. In older adults undergoing PLIF, clinically important medical events may not be confined to the inpatient stay but may also emerge shortly after discharge during the immediate recovery period. Restricting the endpoint to in-hospital events alone could therefore underestimate clinically relevant postoperative vulnerability.

Beyond association testing, we evaluated the predictive performance of the preoperative Age + mFI-5 model. We deliberately favoured a parsimonious model specification to match the modest event count and to preserve bedside usability. The final preoperative model included only two predictors, age and mFI-5, yielding an events-per-variable ratio that was considered acceptable for this dataset, although external validation remains necessary. The model demonstrated fair-to-good discrimination (AUC ~ 0.75). Bootstrap-based internal validation showed only modest optimism, with the AUROC decreasing from 0.744 to 0.734 after correction. Likewise, the optimism-corrected calibration intercept remained close to 0, and the calibration slope remained close to 1, suggesting that the parsimonious Age + mFI-5 model was not substantially overfitted in this dataset. The predicted risk heatmap further illustrated the complementary contributions of age and frailty burden: higher mFI-5 shifted the high-risk boundary to substantially younger ages, whereas patients with mFI-5 = 0 crossed the same decision threshold only at very advanced age. Practically, this reinforces that frailty captures dimensions of vulnerability not fully explained by chronological age alone while also highlighting that extreme age can confer substantial risk even in the absence of measured comorbidity burden. Nevertheless, external validation is still required before broader clinical implementation.

An important issue is the generalizability of the present findings. This study was conducted at a single tertiary centre, and all procedures were performed by one of three fellowship-trained spine surgeons using a standardized PLIF technique. Such procedural consistency likely reduced technical heterogeneity and strengthened internal validity. However, these same features may limit transportability to centres with different operative workflows, perioperative pathways, transfusion thresholds, discharge practices, and patient case-mix profiles. Moreover, our cohort reflects a specific population of older Korean adults undergoing elective PLIF for degenerative lumbar disease, with a mean age of 72.6 years and a relatively modest BMI distribution. Because demographic structure, comorbidity prevalence, frailty burden, and perioperative care patterns may differ across international spine populations, the absolute risk estimates and threshold-based interpretation of the Age + mFI-5 model should be applied cautiously outside similar settings.

Several limitations warrant consideration. First, this was a single-centre retrospective analysis, which may limit generalisability and is susceptible to selection bias and residual confounding. Second, although the Age + mFI-5 model showed reasonable performance in this cohort, it was evaluated internally and still requires independent external validation before broader implementation. Third, mFI-5 is a pragmatic comorbidity-based tool and does not directly measure important frailty domains such as sarcopenia, gait speed, grip strength, nutritional status, or cognitive vulnerability, factors that may further refine risk stratification in older spine patients. Fourth, postoperative events can co-occur within the same patient, and time-agnostic endpoint definitions may obscure causal ordering; thus, postoperative variables such as transfusion, SSI, and reoperation should be interpreted primarily as course markers rather than baseline causal predictors. Fifth, although all PLIF procedures were performed using a standardized technique by experienced spine surgeons, the operating team was not identical in every case. Such heterogeneity may have influenced operative workflow and perioperative outcomes. Sixth, by combining in-hospital and 30-day post-discharge medical complications into a single endpoint, we may have introduced heterogeneity in event timing and mechanism, although this approach was intended to better capture the overall burden of early postoperative medical morbidity in older adults. Although a time-to-event approach might provide additional temporal resolution, the present study was designed primarily to evaluate the occurrence of clinically relevant early postoperative medical complications within a predefined 30-day window rather than to model event timing itself.

Future study should focus on prospective, multi-centre validation of the proposed preoperative risk model, incorporation of broader frailty and sarcopenia metrics, and evaluation of clinical utility using decision-analytic frameworks such as decision curve analysis. Such efforts could enable not only more accurate risk prediction but also targeted prehabilitation and perioperative optimization strategies aimed at reducing medical complications in high-risk older adults undergoing PLIF.

## 5. Conclusions

In older adults undergoing PLIF for degenerative lumbar disease, preoperative frailty quantified by the mFI-5 and chronological age were independently associated with postoperative medical complications. Patients with moderate-to-severe frailty (mFI-5 ≥ 2) experienced a markedly higher burden of medical adverse events, supporting the value of frailty assessment for preoperative risk stratification and perioperative planning. A simple preoperative model combining age and mFI-5 demonstrated fair-to-good discriminative performance with acceptable calibration, providing an interpretable framework to identify high-risk patients and to guide counselling, resource allocation, and tailored perioperative optimization. Further external validation and prospective studies incorporating objective frailty and sarcopenia measures are warranted to refine prediction and improve outcomes in this growing surgical population.

## Figures and Tables

**Table 1:** Items included in mFI-5.

1. Diabetes mellitus: non-insulin, insulin, oral
2. Congestive heart failure within 30 days before surgery
3. Hypertension requiring medication
4. History of COPD (chronic obstructive pulmonary disease) or pneumonia
5. Functional health status before surgery: partially dependent or totally dependent

The table lists the five variables comprising the modified Frailty Index-5 (mFI-5).

**Table 2:** Comparison of patient characteristics according to the presence of medical complications.

Characteristic	No CxME (*n* = 202)	CxME (*n* = 53)	*p*-Value
**Demographics**			
Age (years)	71.92 ± 4.70	75.23 ± 4.59	***<0.001***
Sex (M:F)	87:115	27:26	0.305
BMI (kg/m^2^)	24.62 ± 3.08	24.73 ± 3.76	0.985
Current Smoker	13 (6.4%)	4 (7.5%)	0.760
Alcohol Use	20 (9.9%)	3 (5.7%)	0.428
**Preoperative Characteristics**			
Hypertension	142 (70.3%)	42 (79.2%)	0.196
Diabetes Mellitus	46 (22.8%)	26 (49.1%)	***<0.001***
Dyslipidemia	26 (12.9%)	10 (18.9%)	0.264
Chronic Kidney Disease	7 (3.5%)	2 (3.8%)	1.000
COPD	5 (2.5%)	3 (5.7%)	0.369
Coronary Artery Disease	29 (14.4%)	7 (13.2%)	0.831
Congestive Heart Failure	2 (1.0%)	2 (3.8%)	0.192
Cerebrovascular Accident	9 (4.5%)	0 (0.0%)	0.211
Tuberculosis	4 (2.0%)	2 (3.8%)	0.607
Dependency	28 (13.9%)	15 (28.3%)	***0.012***
ASA Physical Status			0.259
1	6 (3.0%)	0 (0.0%)	
2	148 (73.3%)	35 (66.0%)	
3	47 (23.3%)	18 (34.0%)	
4	1 (0.5%)	0 (0.0%)	
mFI-5 Score			***<0.001***
0	43 (21.3%)	7 (13.2%)	
1	104 (51.5%)	11 (20.8%)	
2	46 (22.8%)	27 (50.9%)	
3	9 (4.5%)	8 (15.1%)	
Preoperative Hemoglobin (g/dL)	13.05 ± 1.42	12.65 ± 1.38	0.069
Femur Neck BMD (T-score)	−1.27 ± 0.98	−1.29 ± 1.26	0.606
Spine BMD (T-score)	−0.81 ± 1.54	−0.74 ± 1.74	0.949
Antiplatelet Agent Use	43 (21.3%)	16 (30.2%)	0.236
Anticoagulant Use	10 (5.0%)	5 (9.4%)	0.206
**Surgery-related Variables**			
Fusion Levels			0.759
1	117 (59.4%)	30 (56.6%)	
2	65 (33.0%)	20 (37.7%)	
≥3	15 (7.6%)	3 (5.7%)	
Operation Time (min)	261.41 ± 62.84	264.62 ± 56.66	0.336
Transfusion	24 (11.9%)	15 (28.3%)	***0.005***
Hemoglobin Drop (g/dL)	2.14 ± 1.11	1.95 ± 1.14	0.226
**Postoperative Variables**			
Surgical Site Infection	5 (2.5%)	7 (13.2%)	***0.004***
Surgical Complications	24 (11.9%)	10 (18.9%)	0.183
Reoperation	9 (4.5%)	7 (13.2%)	***0.028***
Length of Stay (days)	11.96 ± 7.67	24.94 ± 31.53	***<0.001***
Mortality	0 (0.0%)	3 (5.7%)	***0.009***

Demographic characteristics, preoperative clinical variables, surgery-related factors, and postoperative outcomes are compared between patients with and without postoperative medical complications (CxME). Continuous variables are presented as mean ± standard deviation and categorical variables as number (percentage). Medical complications are defined as Clavien–Dindo grade II or higher events.

**Table 3:** Comparison of patient characteristics according to the modified Frailty Index-5.

**Characteristic**	**mFI-5 = 0 (*n* = 50)**	**mFI-5 = 1 (*n* = 115)**	**mFI-5 ≥ 2 (*n* = 90)**	***p*** **-Value**
**Demographics**				
Age (years)	71.14 ± 4.74	72.56 ± 4.72	73.49 ± 4.94	***0.029***
Sex (M:F)	20:30	45:70	49:41	0.069
BMI (kg/m^2^)	23.80 ± 2.77	24.76 ± 3.09	24.97 ± 3.56	0.239
Current Smoker	6 (12.0%)	5 (4.3%)	6 (6.7%)	0.194
Alcohol Use	5 (10.0%)	11 (9.6%)	7 (7.8%)	0.874
**Preoperative Characteristics**				
Hypertension	0 (0.0%)	98 (85.2%)	86 (95.6%)	***<0.001***
Diabetes Mellitus	0 (0.0%)	7 (6.1%)	65 (72.2%)	***<0.001***
Dyslipidemia	3 (6.0%)	13 (11.3%)	20 (22.2%)	***0.015***
Chronic Kidney Disease	0 (0.0%)	2 (1.7%)	7 (7.8%)	***0.021***
COPD	0 (0.0%)	0 (0.0%)	8 (8.9%)	***<0.001***
Coronary Artery Disease	1 (2.0%)	15 (13.0%)	20 (22.2%)	***0.004***
Congestive Heart Failure	0 (0.0%)	0 (0.0%)	4 (4.4%)	***0.024***
Cerebrovascular Accident	0 (0.0%)	5 (4.3%)	4 (4.4%)	0.320
Tuberculosis	0 (0.0%)	2 (1.7%)	4 (4.4%)	0.211
ASA Physical Status 1	6 (11.8%)	0 (0.0%)	0 (0.0%)	***<0.001***
2	39 (76.5%)	99 (86.1%)	45 (50.6%)	
3	6 (11.8%)	16 (13.9%)	43 (48.3%)	
4	0 (0.0%)	0 (0.0%)	1 (1.1%)	
Preoperative Hemoglobin (g/dL)	13.41 ± 1.29	12.98 ± 1.27	12.69 ± 1.59	***0.016***
Femur Neck BMD (T-score)	−1.11 ± 0.83	−1.35 ± 1.05	−1.28 ± 1.14	0.246
Spine BMD (T-score)	−0.94 ± 1.46	−0.76 ± 1.63	−0.76 ± 1.60	0.618
Antiplatelet Agent Use	3 (5.9%)	24 (20.9%)	32 (36.0%)	***<0.001***
Anticoagulant Use	1 (2.0%)	2 (1.7%)	12 (13.3%)	***<0.001***
Dependency	0 (0.0%)	15 (13.0%)	21 (23.6%)	***<0.001***
**Surgery-related Variables**				
Fusion Level				0.232
1	29 (58.0%)	66 (57.4%)	52 (57.8%)	
2	16 (32.0%)	39 (33.9%)	33 (36.7%)	
≥3	5 (10.0%)	10 (8.7%)	5 (5.6%)	
Operation Time (min)	258.20 ± 56.20	266.43 ± 65.35	258.67 ± 59.54	0.788
Transfusion	3 (5.9%)	15 (13.0%)	21 (23.6%)	***0.007***
Hemoglobin Drop (g/dL)	2.27 ± 1.04	2.16 ± 1.05	1.93 ± 1.22	0.056
Estimated Blood Loss (mL)	374.24 ± 205.44	384.44 ± 299.04	317.01 ± 284.77	***0.046***
**Postoperative Indices**				
Medical Complications	7 (14.0%)	11 (9.6%)	35 (38.9%)	***<0.001***
Clavien-Dindo Classification I	44 (86.3%)	103 (89.6%)	55 (61.8%)	***<0.001***
II	7 (13.7%)	11 (9.6%)	30 (33.7%)	
III	0	0	0	
IV	0	0	2 (2.2%)	
V	0	1 (0.9%)	2 (2.2%)	
Surgical Site Infection	2 (4.0%)	7 (6.1%)	3 (3.3%)	0.630
Surgical Complications	3 (6.0%)	18 (15.7%)	13 (14.4%)	0.228
Reoperation	2 (4.0%)	9 (7.8%)	5 (5.6%)	0.610
Readmission	2 (4.0%)	9 (7.8%)	4 (4.4%)	0.486
Length of Stay (days)	11.66 ± 8.31	14.36 ± 15.89	16.70 ± 20.56	***<0.001***
Mortality	0 (0.0%)	1 (0.9%)	2 (2.2%)	0.464

Demographic characteristics, preoperative clinical variables, surgery-related factors and postoperative outcomes stratified by mFI-5 category (0, 1, and ≥2). Data are presented as mean ± standard deviation or number (percentage). *p*-values represent comparisons across frailty groups.

**Table 4:** Multivariable logistic regression analysis of factors associated with medical complications.

Predictor	OR (95% CI)	*p*-Value
**Age (per 1-year increase)**	1.133 (1.055–1.216)	**<0.001**
**mFI-5 (per 1-point increase)**	2.103 (1.387–3.188)	**<0.001**

Multivariable logistic regression analysis identifying independent predictors of postoperative medical complications. Results are reported as odds ratios (ORs) with 95% confidence intervals (CIs). Age and mFI-5 were analyzed as continuous variables.

## Data Availability

The data presented in this study are available upon request from the corresponding author. The data are not publicly available due to privacy and ethical restrictions.

## Supplementary Materials

The following supporting information can be downloaded at: https://www.mdpi.com/article/10.3390/jcm15082847/s1, Table S1: Performance of the Youden-optimal probability cut-off; Table S2: Clinically grouped details of postoperative medical complications (CxME).
