# Supplementary material for: Frailty (mFI-5) and Age Predict Medical Complications After Posterior Lumbar Interbody Fusion in Older Adults: A Retrospective Cohort Study
Source: J Clin Med. 2026 Apr 9;15(8):2847. doi: 10.3390/jcm15082847 (PMC13115792; doi:10.3390/jcm15082847)
Supplement: Supplementary file 1 [file jcm-15-02847-s001.zip › jcm-4169771-supplementary.pdf]

Supplementary Table S1. Performance of the Youden-optimal probability cut-off

Model: Age + mFI-5 logistic regression. N=255; events (CxME=1)=53. Cut-off p=0.222 (predict CxME if p ≥ cut-off).

Cut-off (p)	Sensitivity	Specificity	PPV	NPV	Accuracy
0.222	0.717	0.723	0.404	0.907	0.722

Youden index (J) = 0.440.

Confusion matrix

	Predicted CxME=1	Predicted CxME=0
Observed CxME=1	38	15
Observed CxME=0	56	146

Performance metrics of the Youden-optimal probability cut-off for the Age + mFI-5 logistic regression model predicting postoperative medical complications(CxME). Sensitivity, specificity, positive predictive value(PPV), negative predictive value(NPV), accuracy, Youden index, and the corresponding confusion matrix are presented.

Supplementary Table S2. Clinically grouped details of postoperative medical complications (CxME)

**CxME category**	**n**	**% of patients with CxME (n=53)**
Urinary tract infection / suspected UTI	19	35.8
Pulmonary complication or infection	9	17.0
Fever of unknown or unclear source	7	13.2
Delirium / altered mental status	6	11.3
Cardiovascular or thromboembolic event	4	7.5
Neurologic event	4	7.5
Urinary retention / lower urinary tract issue	3	5.7
Acute kidney injury	3	5.7

CxME category	n	% of patients with CxME (n=53)
Gout flare	3	5.7
Other infection requiring treatment	2	3.8
Endocrine / metabolic event	2	3.8
Hepatobiliary / laboratory abnormality	2	3.8
Procedure-related peripheral complication	1	1.9

Complication details were extracted from free-text records and grouped into clinically meaningful categories for supplementary presentation. Because some patients experienced more than one medical complication, percentages do not sum to 100%.
